# Homochiral Metal-Organic Framework Based Mixed Matrix Membrane for Chiral Resolution

**DOI:** 10.3390/membranes12040357

**Published:** 2022-03-24

**Authors:** Hwa-Jin Choi, Dong-Yeun Koh

**Affiliations:** Department of Chemical and Molecular Engineering (BK-21 Four), Korea Advanced Institute of Science and Technology, Daejeon 34141, Korea; hwajinchoi@kaist.ac.kr

**Keywords:** Mixed Matrix Membrane, Torlon®, Metal-Organic Framework, enantioselectivity

## Abstract

Efficient separation of enantiomers is critical in the chemical, pharmaceutical, and food industries. However, conventional separation methods, such as chromatography, crystallization, and enzymatic kinetic resolution, require high energy costs and specific reaction conditions for the efficient purification of one enantiomer. In contrast, membrane-based processes are continuous processes performed with less energy than conventional separation processes. Enantioselective polymer membranes have been developed for the chiral resolution of pharmaceuticals; however, it is difficult to generate sufficient enantiomeric excess (ee) with polymer membranes. In this work, a homochiral filler of L-His-ZIF-8 was synthesized by the ligand substitution method and mixed with polyamide(imide) (i.e., Torlon^®^) to fabricate an enantioselective mixed-matrix membrane (MMM). The enantio-selective separation of R-1-phenylethanol over S-1-phenylethanol was demonstrated with a 25 wt% loaded L-His-ZIF-8/Torlon^®^ MMM in an organic solvent nanofiltration (OSN) mode.

## 1. Introduction

In the pharmaceutical, life science, and food industries, the chirality of compounds has a vital role in enzymatic reactions to obtain the targeted effect [1,2]. Conventional separation processes such as chromatography, crystallization, and enzymatic kinetic resolution have been shown to resolve pure enantiomers effectively [3]. However, traditional methods have difficulties in the scaled-up operation of separation processes [4]. For example, enzymatic kinetic resolution, which depends on a catalytic reaction, has a disadvantage in that the activity of the catalyst decreases over time [5]. Many chiral molecules are resolved using chiral stationary phases (CSPs) in the form of high-performance liquid chromatography (HPLC) [1,6]. In particular, polymer-based CSPs are commonly adopted for chiral separations due to their porous structure and diverse surface functionalities [7,8,9]. Membrane-based separations have recently appeared as low-energy processes for large-scale and continuous operations in the pharmaceutical industry. Membrane-based separation generally has distinct advantages: low energy consumption, solution processability of material, large specific surface area, and tunable pore structure of the membranes. Recently, organic solvent nanofiltration (OSN) has emerged as a new technology to improve sustainability in the pharmaceutical industry. In this work, we demonstrate the enantioselective separation of OSN-based membrane processes.

Various membrane materials have been investigated for the scalable separation of solvent–solute pairs [10]. Solution processability of polymeric materials enables the straightforward production of polymeric membranes in various forms (e.g., films and hollow fibers) at large scales. However, polymeric membranes are typically unstable in organic solvents and exhibit a low permeation flux or separation capacity [11]. Although many researchers have strived to optimize the membrane fabrication condition or develop new polymeric materials to improve the separation performance of polymeric membranes, conventional polymeric membranes generally cannot exceed Robeson’s upper bound. The upper bound can often be overcome using a membrane fabricated with microporous materials, such as a carbon molecular sieve (CMS), metal-organic frameworks (MOFs), and zeolite [12]. These microporous materials can also be incorporated into the polymeric matrix to form mixed-matrix membranes (MMMs) that boost the intrinsic performances of the composite membranes. MMMs have demonstrated outstanding performance as a new class of membrane that combines the advantages of a superior molecular sieving performance, diverse functionalities of the inorganic fillers and solution-processability of the organic matrices [10,11,13]. However, simple molecular sieving properties of the inorganic fillers cannot be fully used in enantiomeric separations. Two enantiomers cannot be separated through the same molecular sieve membranes because the sizes of the two enantiomers are identical to each other. The enantioselective MMMs must have chiral recognition sites to separate the enantiomers, and incorporating chiral fillers can induce this chirality in the composite membrane. This work focused on the fabrication of an enantioselective MMM based on homochiral metal-organic frameworks (MOFs).

MOFs are an emerging class of porous materials combining various ligands and center metals to enable the separation of a wide variety of molecular mixtures [14]. Homochiral MOFs have garnered tremendous attention because of their potential for enantiomer separation, although, as noted, enantiomeric separation cannot be achieved via simple molecular sieving. Instead, the pores can be tuned to provide chiral environments for enantiomeric mixtures [12]. A chiral MOF, a (R)-CuMOF-1–silica composite, has a strong binding affinity to a single type of enantiomer and was utilized as a chiral stationary phase in HPLC to separate enantiomeric mixtures including racemic sulfoxides, *sec*-alcohols, *β*-lactams, benzoins, and flavanones epoxides [15]. TAMOF-1 is another chiral MOF that enables (±)-ibuprofen and (±)-thalidomide separation in a packed column [16]. The chiral stationary phase in the HPLC column must meet the application-specific resolution of enantiomers, and some reported chiral MOFs are plausible candidates [17,18].

There are enantioselective MMMs manufactured based on such homochiral MOFs. For instance, Lu et al. discovered that MIL-53-NH-L-His/PES for chiral separations of 1-phenylethanol enantiomers [19].

**Figure 1:**
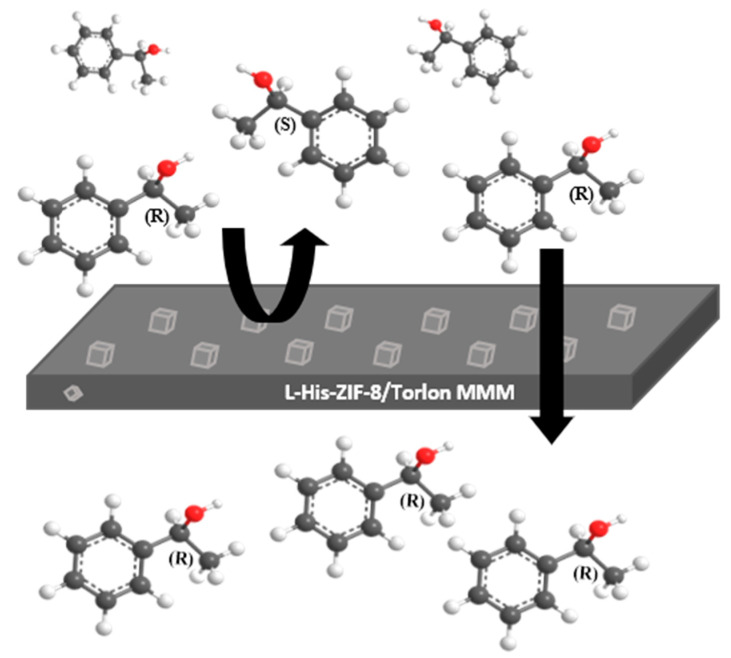
Scheme of the racemic 1-phenylethanol resolution in the L-His-ZIF-8/Torlon^®^ mixed-matrix membrane.

In this work, the chiral filler *L-*His-ZIF-8, synthesized via ligand substitution, was incorporated into polyamide(imide) (i.e., Torlon^®^) to fabricate enantioselective mixed-matrix membranes. Torlon^®^ was chosen because of its high solvent resistance and excellent stability for experiments with organic solvent. ZIF-8 has been shown to have superior thermal stability, large surface area and permanent porosity with aperture size (3.4 Å) [16]. In addition, ZIF-8 showed high compatibility with polyimides when fabricated into a mixed matrix membrane due to the organic property of the imidazolate linker [20]. Based on these advantages, this ZIF-8 was altered to a chiral molecule that enables chiral resolution through the ligand substitution method with *L*-histidine. Chiral L-histidine could be substituted because it has a functional group similar to 2-methylimidazole, a ligand of ZIF-8. In other words, altered frameworks are designed to have different interactions with chiral molecules to separate them. As well as the chiral property, it is expected that organic properties will be increased by inserting *L*-histidine into the ZIF-8 framework, which can improve the compatibility with the Torlon^®^ polymer. In this work, a mixed matrix membrane using *L*-His-ZIF-8 and Torlon^®^ for separating an enantiomer was tested. It was proved that there is resolution by using only the L-His-ZIF-8 [21], and in our experiment, the resolution was observed by fabricating it in the form of an MMM. As shown in Figure 1, (±)-1-phenylethanol was the target molecule to prove the chirality of the mixed-matrix membrane. The *R*-enantiomer of 1-phenylethanol is a useful chemical as an ophthalmic preservative, as an inhibitor of cholesterol adsorption, and as a component of fragrances [20].

## 2. Materials and Methods

### 2.1. Materials

Zinc nitrate hexahydrate (Zn(NO_3_)_2_∙6H_2_O, 98%, Sigma Aldrich, Saint Louis, MI, USA), 2-methylimidazole (Hmim, 99%), triethylamine (TEA, 99%), L-histidine (L-His, 99%, Sigma Aldrich, Saint Louis, MI, USA), Methyl Alcohol (Methanol) and 1-methyl-2-pyrrolidinone (NMP, 99%, TCI) were used in this study. Acetic Acid-D4 (99.5%) was obtained from Cambridge, and Ethyl Alcohol (Ethanol, HPLC) and n-hexane (99.5%) were obtained from Daejung chemicals and metals (Daejeon, Korea). Trifluoracetic acid (TFA, HPLC) and the racemates (±)-1-phenylethanol (97%) were obtained from Alfa Aesar. All solvents and chemicals were of reagent quality and used without further purification.

### 2.2. Membrane Fabrication

#### 2.2.1. Synthesis of Microporous L-His-ZIF-8 in Methanol

The synthesis of L-his-ZIF-8 was according to that of Yu et al. [22]. L-histidine was first dissolved in deionized water followed by the addition of triethylamine into the solution. Additionally, methanol was added to the L-histidine/H_2_O solution. Subsequently, zinc nitrate hexahydrates and 2-methylimidazole were separately dissolved in the solution. Then, the separated solutions were mixed. The molar ratio of L-histidine and 2-methylimidazole is about 8:1. After stirring for 24 h at room temperature, the solution was collected by centrifugation, followed by methanol washing. Finally, the obtained white powder was dried at 120 °C.

#### 2.2.2. Preparation of the Torlon^®^ Dense Membrane

Torlon^®^ 4000T was dried in a vacuum oven at 120 °C overnight before use to remove any remaining water. After drying, Torlon^®^ 400T/NMP casting dope was prepared by dissolving Torlon^®^ to the NMP. The glass plate was thoroughly washed with acetone and deionized water followed by drying in an oven at 120 °C. Then, the dope solution was casted using a blade with thickness of 50 μm on the glass plate at room temperature and dried for 2 days at 45 °C to remove any residual solvent. Lastly, the casted membrane was washed with pure water. Once the film was formed, it could be easily separated from the glass plate in pure water. Finally, after soaking the membrane in methanol and hexane, it was dried in a vacuum oven at 120 °C overnight.

#### 2.2.3. Preparation of the Mixed Matrix Membrane

MMMs containing different L-His-ZIF-8 loadings (15, 20, 25 wt%) were fabricated using the prime method. Two solutions of polymer and L-His-ZIF-8 were prepared in two separate vials. For the prime method, a 10 wt% Torlon^®^ solution was prepared. Then, 15, 20 and 25 wt% of L-His-ZIF-8 crystals were dispersed in the NMP. Subsequently, the L-His-ZIF-8 solution was primed by the 10 wt% Torlon^®^ solution, which was further mixed by Voltex. Following in-depth mixing, the solid-state Torlon^®^ polymer was added, and the mixture was thoroughly mixed by a jar roller for 1 day. Finally, the polymer and filler dope solution was casted onto the glass plate at room temperature and dried for 2 days at 45 °C to remove any residual solvents. Again, the casted membrane was washed with pure water, methanol and hexane and dried in a vacuum oven at 120 °C overnight.

### 2.3. Characterization

#### 2.3.1. L-His-ZIF-8

Attenuated Total Reflectance Fourier Transform Infrared Spectroscopy (ATR-FTIR) analysis was performed on a Thermo Fisher Scientific Instrument Nicolet iS50 (Walttham, MA, USA) spectrometer at room temperature with a wavenumber range of 4000–400 cm^−1^ to determine the change with the ZIF-8 functionality. To obtain the crystal size of the L-His-ZIF-8, scanning electron microscopy (FEI Magellan400) was performed. Powder X-ray diffraction (RIGAKU, SmartLab) measurements were done on the powder at room temperature. The Circular Dichroism Spectropolarimeter (Jasco-815-150-L, Jasco Inc., Easton, PA, USA) was used to identify the chirality of the L-His-ZIF-8 with a wavenumber range of 220–380 nm. 1H NMR spectrum of the L-His-ZIF-8 (600 MHz, CD3COOD) was obtained to calculate the ratio of the ligand.

#### 2.3.2. L-His-ZIF-8/Torlon^®^ Mixed Matrix Membrane

Powder X-ray diffraction (RIGAKU, SmartLab) measurements were done on pristine Torlon^®^, L-His-ZIF-8 and L-His-ZIF-8/Torlon^®^ MMM at room temperature to check the incorporation of the filler in the MMMs. Scanning electron microscopy (FEI Magellan400) was performed to obtain the surface and cross-section morphology of the Torlon^®^ membrane and mixed matrix membrane. To check whether the L-His-ZIF-8 crystal is well integrated into the MMM, L-His-ZIF-8/Torlon^®^ was characterized with element mapping by energy-dispersive X-ray spectroscopy (EDS).

#### 2.3.3. Organic Solvent Nanofiltration (OSN) Experiments

To confirm the resolution of the L-His-ZIF-8, an experimental group and a control group were established. The experimental group was the MMM mixed with L-hi-ZIF-8 and Torlon^®^, and the control group was a membrane using only Torlon^®^ without L-His-ZIF-8. The OSN test of the pristine Torlon^®^ membrane was performed with HP 4750 dead-end filtration at 60 bar, 80 rpm and room temperature, to evaluate the permeance of the organic solvent and the separation of (±)-1-phenylethanol with molecular weights of 122.16 g/mol. In the case of the 25 wt% L-His-ZIF-8/Torlon^®^ MMM, the experiment was conducted at 50 bar, and all other conditions were the same. The active area of the L-His-ZIF-8/Torlon^®^ MMM was about 14.6 cm^2^.

(1) $$P=\frac{V}{A\times t\times ∆p}$$

V: permeate volume, A: membrane’s area, t: unit time, ∆p: transmembrane pressure.

**Figure 2:**
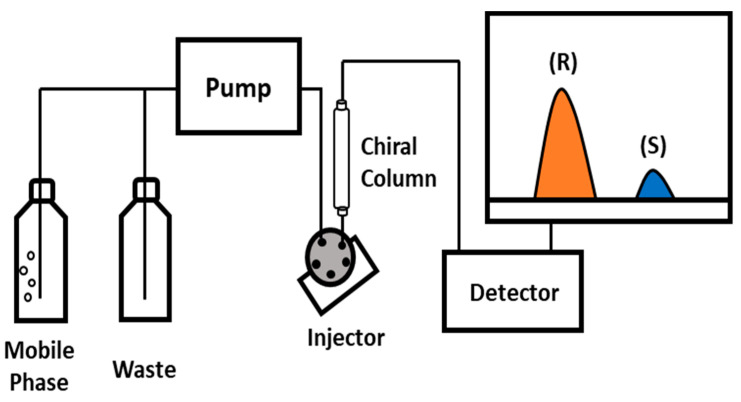
High Performance Liquid Chromatography to analyze the composition of the permeate. Racemic compounds were separated using a mixed solvent of Hexane/Ethanol/TFA (90:10:0.1, *v*/*v*). The UV monitor wavelength was 254 nm.

The permeate after the OSN test was injected to calculate the area ratio of the R/S. Figure 2 shows the UV/vis detector set to a wavelength of 254 nm and a DAICE CHIRALPAK IG (250 × 4.6 mm) column in the YL9100 HPLC system. All solvents for the HPLC Mobile Phase were as follows: 0.1% Trifluoro acetic acid in n-Hexane/EtOH solvent mixture (95:5 *v*/*v*). The area value is calculated based on the HPLC data, and the enantiomeric excess could finally be calculated. As can be seen from the equation for calculating the enantiomeric excess, it is an indicator that represents the ratio of each enantiomer, that is, the enantiomeric separation ability.

(2) $$\%\;ee=\frac{\;\left|R-S\right|\;}{R+s}\times 100$$

*ee*: enantiomeric excess, R Area of R enantiomer, S: Area of S enantiomer.

## 3. Results and Discussion

### 3.1. Chiral Filler: L-His-ZIF-8

**Figure 3:**
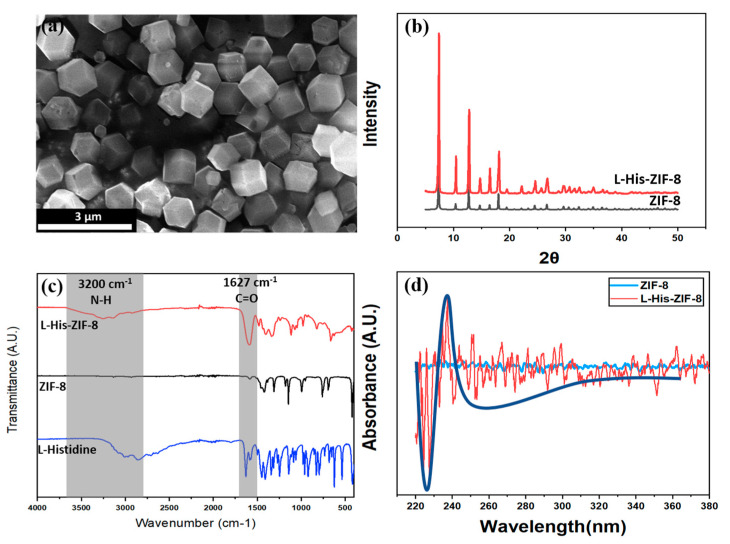
Characterization of the Synthesized L-His-ZIF-8: (**a**) SEM Images of the synthesized L-His-ZIF-8. (**b**) PXRD patterns of ZIF-8 and L/D-His-ZIF-8. (**c**) FTIR Spectra of L-His-ZIF-8, ZIF-8, and L-histidine, respectively. (**d**) Circular Dichroism spectra of ZIF-8 and L-His-ZIF-8.

**Figure 4:**
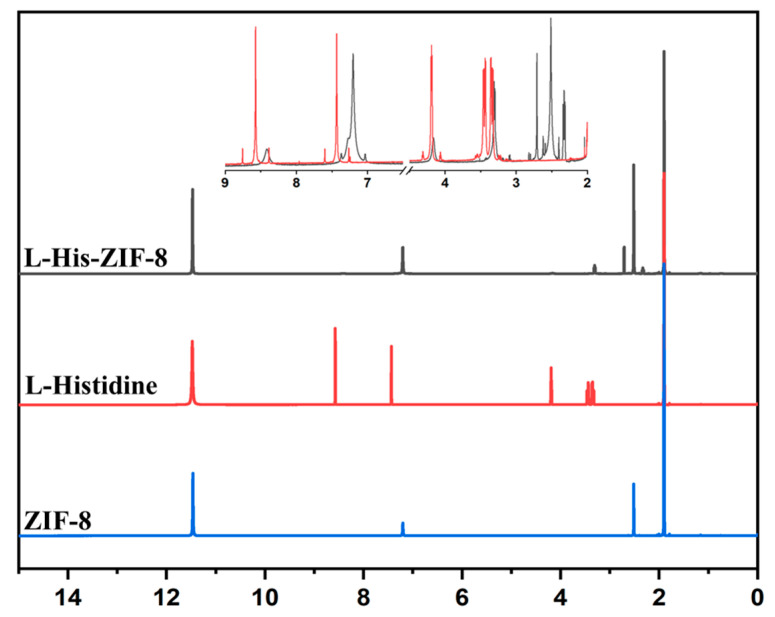
1H NMR Spectrum (600 MHz, CD3COOD) of the ZIF-8, L-Histidine and L-His-ZIF-8.

As shown in Figure 3a, it was confirmed that L-His-ZIF-8 was synthesized with a uniform size of 1 µm. At the same time, it is necessary to confirm whether L-histidine is inserted into the existing ZIF-8 lattices. As mentioned earlier, L-His-ZIF-8 was synthesized by ligand substitution replacing the ligand in the existing framework. Powder X-ray diffraction (PXRD) shows the L/D-His-ZIF-8 has the same structure as the pristine ZIF-8 based on the same position of the peak (Figure 3b). Typical peaks were observed for ZIF-8 with 2θ at 7.48, 10.48, 12.88, 14, 16.4 and 18 [23]. The L/D-His-ZIF-8 also has a sodalite (SOD) topology like the ZIF-8 architecture. Attenuated Total Reflectance Fourier Transform Infrared Spectroscopy (ATR-FTIR) analysis confirmed that the synthesized L-His-ZIF-8 contained L-histidine molecules by confirming the adsorption peak at 1627 cm^−1^ assigned to the C=O stretch in the carboxy group for L-histidine (Figure 3c). Additionally, the broad adsorption peaks between 3600 and 2700 cm^−1^ are attributed to the N-H groups and O-H stretching vibrations together with the C-H stretching bonds. Thus, it can be acknowledged that the L-histidine molecule was inserted into a part of the existing structure of the ZIF-8. As shown in Figure 4, the ratio of 2-Hmim to L-Histidine was calculated to be 9:1 by the ^1^H NMR Spectrum of L-His-ZIF-8 (600 MHz, CD_3_COOD). Circular Dichroism Spectropolarimetry was performed to characterize a homochirality of the synthesized L-His-ZIF-8 (Figure 3d). Compared with pure ZIF-8, an obvious adsorption peak at 235 cm^−1^ demonstrating the chiral L-histidine molecule successfully substituted part of the ZIF-8. 

### 3.2. Enantioselective L-His-ZIF-8/Torlon^®^

**Figure 5:**
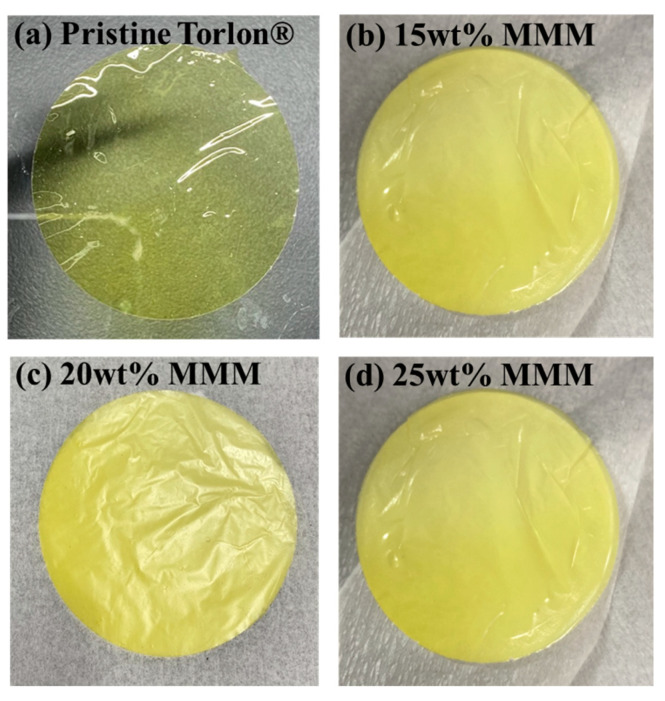
The casted membrane with a doctor blade: (**a**) Image of pristine Torlon^®^ membrane; (**b**) 15 wt% L-His-ZIF-8/Torlon^®^ MMM; (**c**) 20 wt% L-His-ZIF-8/Torlon^®^ MMM; (**d**) 25 wt% L-His-ZIF-8/Torlon^®^ MMM.

**Figure 6:**
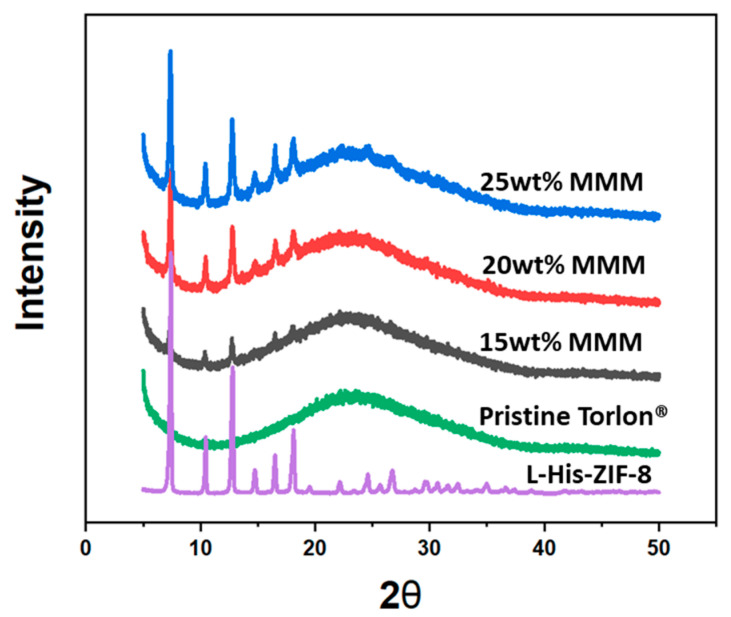
Thin Film X-ray diffraction of the fabricated L-His-ZIF-8/Torlon^®^ Mixed Matrix Membrane compared to the pristine Torlon^®^ dense membrane and L-His-ZIF-8 filler.

**Figure 7:**
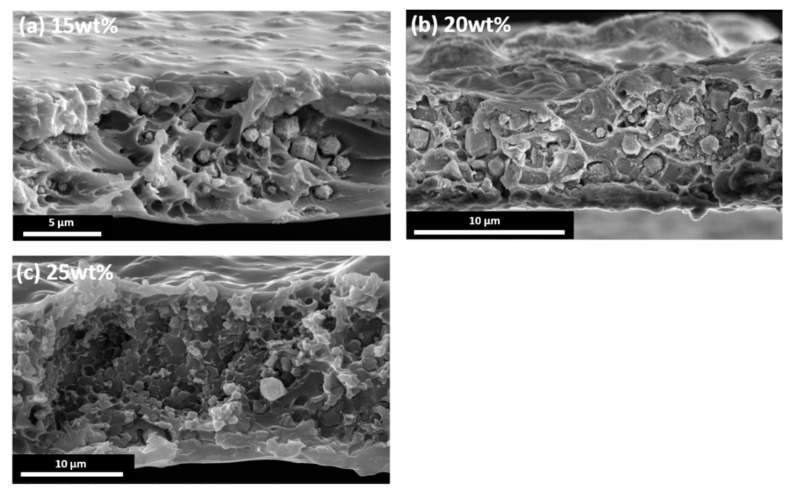
SEM cross-sectional image of the L-His-ZIF-8/Torlon^®^ MMM (**a**) 15 wt% L-His-ZIF-8, (**b**) 20 wt% L-His-ZIF-8, (**c**) 25 wt% L-His-ZIF-8.

**Figure 8:**
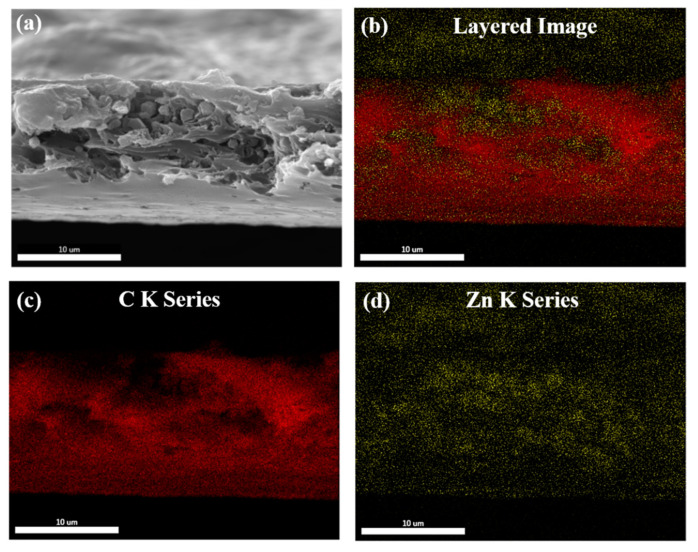
Energy-dispersive X-ray spectroscopy (EDS) of the 20 wt% MMM: (**a**) SEM Image; (**b**) EDS Mapping; (**c**) C Kα (**d**) Zn Kα.

After making the casting dope with various filler/polymer compositions, all MMMs were casted under saturated NMP conditions (Figure 5). Thin Film X-ray Diffraction results supported the incorporation of L-His-ZIF-8 into the MMM (Figure 6). Scanning Electron Microscopy (SEM) was used to demonstrate the cross-section and surface morphology of the membranes (Figure 7). As shown in the image, the thickness of the cross section is about 10 ± 5 μm. The cross-sectional SEM image of the L-His-ZIF-8/Torlon^®^ showed that the fillers were integrated into the polymer interchain. However, it can be seen that an interfacial void was observed especially in the 15 wt% and 20 wt% MMM and, as the filler content was increased, fewer interfacial voids were observed. In general, when the amount of filler escalates in an MMM, the adhesion between the polymer and filler decreases due to agglomeration of the filler which results in an increased “sieve-in-a-cage” morphology. However, L-His-ZIF-8/Torlon^®^ shows the opposite trend. It is presumed that this is due to differences in the membrane fabrication process. In addition, even in the section where the filler was surrounded by Torlon^®^ and the crystal was not distinguished, it can also be proved that the crystal exists in that part by energy dispersive X-ray Spectroscopy (EDS) (Figure 8).

### 3.3. Performance Test via Organic Solvent Nanofiltration

**Figure 9:**
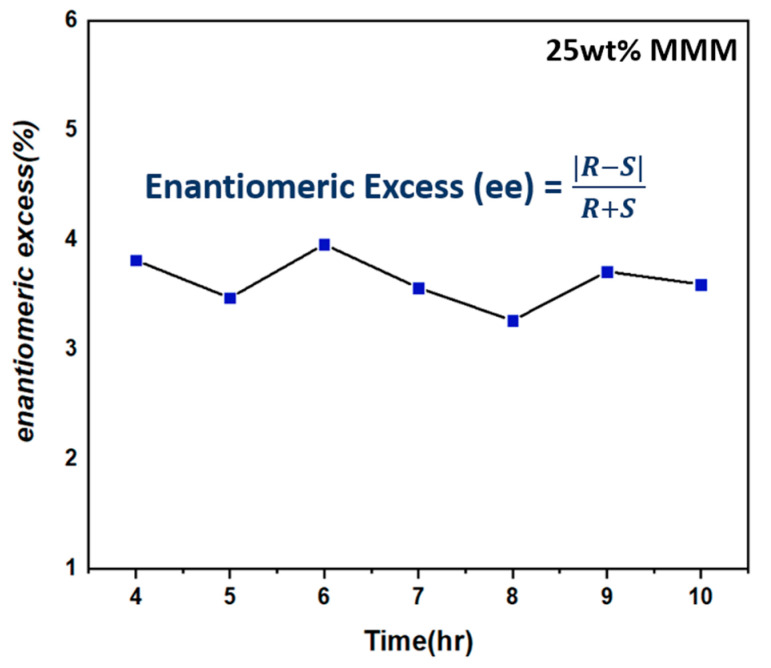
Enantiomeric Excess for (R)-1-phenylehtanol over (S)-1-phenylethanol.

To evaluate the ability to separate a racemic mixture, a Torlon^®^ dense membrane was used to perform the solvent separation with (±)-1-phenylehtanol in a methanol environment. The experiments were performed with a dead-end filtration setup with a transmembrane pressure and temperature of 60 bar and 20 °C, respectively. HPLC measurement was performed to analyze the composition of the permeance. For pristine Torlon^®^ membrane, it was confirmed that there was no difference in the amount of R and S, that is, there was no enantiomeric separation ability by the polymer itself. The methanol permeance for the Torlon^®^ dense membrane was 4.52 × 10^−6^ L m^2^ h bar^−1^. Then the 25 wt% loading L-His-ZIF-8/Torlon^®^ was tested for the enantiomeric separation performance with (±)-1-phenylethanol in methanol. Because the 25 wt% L-His-ZIF-8/Torlon^®^ had the smallest interfacial void among the casted MMMs, it was considered suitable for the performance evaluation. The experiments were performed with the same dead-end filtration setup at a transmembrane pressure and temperature of 50 bar and 20 °C, respectively. As the filler was added into the mixed-matrix membrane, the void space through which the feed could penetrate was increased, and the permeance increased accordingly. HPLC results showed that a difference between the R and S was observed. Using the formula for calculating the enantiomer excess, the ee value for R over S is calculated to be 3.63% (Figure 9). This value is increased by 2.62% compared to the ee value of the pristine Torlon^®^. The permeance for the 25 wt% MMM was 53.84 × 10^−6^ L m^2^ h bar^−1^.

## 4. Conclusions

In this study, enantioselective mixed-matrix membranes were fabricated with the advantages of both a polymer and filler. The chiral filler, L-His-ZIF-8, has a role in the separation of enantiomers in the L-His-ZIF-8/Torlon^®^ MMM. It was confirmed that L-His-ZIF-8 crystals of several hundred nanometers were synthesized, and it could be seen that the chiral ligand was inserted into the ZIF-8 skeletal backbone. To verify the characterization, SEM, XRD, FEIR, CD Spectra and NMR were analyzed. After the filler synthesis, Torlon^®^ was adopted as a polymer for the MMM. Torlon^®^ is a very stable polymer because of its high mechanical strength, thermal stability and chemical resistance. Therefore, Torlon^®^ is a suitable polymer for Organic Solvent Nanofiltration because it does not swell in most organic solvents. MMMs with various contents were manufactured by mixing L-His-ZIF-8 and Torlon^®^. As a result, a 25 wt% MMM was tested to prove the enantioselectivity compared to the Torlon^®^ dense membrane. Torlon^®^ has no selectivity for (±)-1-phenylethanol, and the permeance for the Torlon^®^ dense membrane was 4.52 × 10^−6^ L m^2^ h bar^−1^. In contrast, the enantiomeric excess of the 25 wt% MMM was 3.63%, and the permeance was 53.8 × 10^−6^ L m^2^ hr bar^−1^. The reason for this much larger permeance is attributed to the pore flexibility of the inserted filler.

Considering that the value of *ee* was lower than expected, it is assumed that it is due to the sieve-in-a-cage phenomenon seen in a part of the membrane. This is one of the common problems in MMMs due to the different interactions between the filler and polymer. Once this void is created, the non-selective flow increases, and the performance of the MMM deteriorates. Therefore, if the interaction can be increased, the efficiency is expected to be improved. Additionally, if D-His-ZIF-8 was synthesized and tested to show the opposite trend, the results of this experiment would be further strengthened. Furthermore, the various possibilities that this study presents can be demonstrated by showing that another racemic mixture is also separable. If high efficiency can be achieved, it is expected to improve the economic feasibility of the existing expensive chiral separation process.

## Data Availability

Data available on request.
